# Anti-Inflammatory Potential of Hexane Extract of Mud Lobster (*Thalassina anomala*) in Lipopolysaccharide-Stimulated RAW 264.7 Macrophages

**DOI:** 10.21315/tlsr2021.32.1.9

**Published:** 2021-03-31

**Authors:** Nur Nadiah Zakaria, Masnindah Malahubban, Sharida Fakurazi, Wong Sie Chuong and, Amy Halimah Rajaee

**Affiliations:** 1 Department of Animal Science and Fishery, Faculty of Agricultural Science and Forestry, Universiti Putra Malaysia Bintulu Sarawak Campus, 97000 Bintulu, Sarawak, Malaysia; 2 Department of Science and Technology, Faculty of Humanities, Management and Science, Universiti Putra Malaysia Bintulu Sarawak Campus, 97000 Bintulu, Sarawak, Malaysia; 3 Department of Human Anatomy, Faculty of Medicine and Health Sciences, Universiti Putra Malaysia, 43400 UPM Serdang, Selangor, Malaysia; 4 Halal Products Research Institute, Universiti Putra Malaysia, Putra Infoport, 43400 UPM Serdang, Selangor, Malaysia

**Keywords:** Inflammation, Mud Lobster, Lipopolysaccharide, RAW 264.7 Cells, Keradangan, Udang Lumpur, Lipopolisakarida, Sel RAW 264.7

## Abstract

Mud lobsters are crustaceans from the genus *Thalassina* which are lesser known and seldom seen but are nevertheless an important organism to the mangrove ecosystem. In Malaysia and Thailand, mud lobsters are eaten by locals as treatment for asthma. It is traditionally believed that they are effective in reducing the number of asthma attacks and severity of asthma symptoms. However, the therapeutic potential of mud lobster extract remains unclear and has not been fully elucidated or reported in any scientific study. The objectives of this study are to investigate the anti-inflammatory potential of mud lobster, *Thalassina anomala* extracts (hexane, chloroform and methanol) in lipopolysaccharide (LPS)-stimulated RAW 264.7 macrophages, and to identify the potential bioactive compounds involved. An MTT assay was performed to determine the cytotoxicity of the *T. anomala* extracts on RAW 264.7 macrophages. Nitrite quantification assay and enzyme-linked immunosorbent assay (ELISA) were conducted to investigate the ability of the *T. anomala* extracts to suppress the secretion and expression of nitric oxide (NO), Prostaglandin E_2_ (PGE_2_) and proinflammatory cytokines (TNF-α, IL-6 and IL-1β) in LPS-stimulated macrophages. GC-MS analysis was done to identify putative metabolites. The hexane extract of *T. anomala* showed anti-inflammatory activity by significantly inhibiting the LPS-induced production of NO, PGE_2_, interleukin- (IL-) 6, IL-1β and tumour necrosis factor-alpha (TNF-α) in a concentration-dependent manner. Hexane extract treatment with 100 μg/mL has decreased the NO secretion into 37 μM. Meanwhile, hexane extract at concentration of 100 μg/mL able to significantly suppressed PGE_2_,TNF-α, IL-6 and IL-1β production into 2015 pg/mL, 2406 pg/mL, 460 pg/mL and 9.6 pg/mL, respectively. GC-MS analysis of the hexane extract revealed the presence of 19 putative compounds. The identified compounds were reported to have anti-inflammatory, antioxidant and antibacterial activities. These results suggest that the hexane extract of *T. anomala* potentially has anti-inflammatory properties and concentration dependently suppressed NO, PGE_2_ and proinflammatory cytokines’ production in LPS-stimulated macrophages. The findings provide a rational basis of the traditional use of mud lobster for inflammation-associated ailments.

## Abstract (ms)

Udang lumpur merupakan krustasia daripada genus *Thalassina* yang kurang diketahui dan jarang dilihat namun ia merupakan organisma penting dalam ekosistem paya bakau. Di Malaysia dan Thailand, udang lumpur dimakan oleh penduduk tempatan sebagai penawar kepada asma. Secara tradisional, ia dipercayai berkesan untuk mengurangkan kekerapan serangan asma serta mengurangkan kesan teruk simptom serangan asma. Walau bagaimanapun, potensi terapi ekstrak udang lumpur masih tidak jelas dan belum diterangkan atau dilaporkan sepenuhnya dalam kajian saintifik. Objektif kajian ini adalah untuk mengkaji potensi antikeradangan ekstrak udang lumpur, *Thalassina anomala* (heksana, kloroform dan metanol) terhadap makrofaj RAW 264.7 yang dirangsang dengan lipopolisakarida (LPS) dan mengenal pasti sebatian bioaktif yang berpotensi terlibat. Pencerakinan MTT dilakukan untuk menentukan kesitotoksikan ekstrak *T. anomala* terhadap makrofaj RAW 264.7. Pencerakinan pengkuantitian nitrit dan pencerakinan imunoserap terangkai enzim (ELISA) dijalankan bagi mengkaji keupayaan ekstrak *T. anomala* untuk menyekat perembesan dan pengeluaran nitrik oksida (NO), Prostaglandin E_2_ (PGE_2_) dan sitokin prokeradangan (TNF-α, IL-6 dan IL-1β) dalam makrofaj yang dirangsang dengan LPS. Analisis GC-MS dilakukan untuk mengenal pasti metabolit aktif. Ekstrak heksana *T. anomala* menunjukkan aktiviti antikeradangan dengan merencatkan secara jelas pengeluaran NO, PGE_2_, interleukin- (IL-) 6, IL-1β dan nekrosis tumor faktor-alfa (TNF-α) yang teraruh oleh LPS dalam keadaan yang bergantung kepada kepekatan. Rawatan ekstrak heksana pada 100 μg/mL berupaya mengurangkan perembesan NO menjadi 37 μM. Manakala ekstrak hexana pada kepekatan 100 μg/mL mampu merencatkan pengeluaran PGE_2_, TNF-α, IL-6 dan IL-1β menjadi 2015 pg/mL, 2406 pg/mL, 460 pg/mL dan 9.6 pg/mL. Analisis GC-MS ekstrak heksana menunjukkan kehadiran 19 jenis sebatian andaian. Sebatian yang dikenal pasti dilaporkan mempunyai aktiviti antikeradangan, antioksidan, dan antibakteria. Hasil ini menunjukkan bahawa ekstrak heksana *T. anomala* berpotensi mempunyai sifat antikeradangan dan bergantung kepada kepekatan, menyekat pengeluaran NO, PGE_2_ dan sitokin prokeradangan dalam makrofaj yang dirangsang dengan LPS. Dapatan kajian ini memberikan asas rasional bagi penggunaan udang lumpur secara tradisional untuk penyakit berkaitan keradangan.

**Highlights**

- All samples collected from Kuala Tatau, Bintulu; Kuala Balingian, Mukah and Sarikei were identified as *Thalassina anomala.*
- The hexane extract of *T. anomala* exhibits anti-inflammatory potential in LPS-stimulated RAW 264.7 macrophages.
- The non-polar compounds detected in the hexane extract of *T. anomala* by GC-MS analysis revealed 19 putative metabolites which may contribute to the anti-inflammatory activities.

## INTRODUCTION

Mud lobsters (*Thalassina* spp.) are nocturnal organisms that belong to the Order Decapoda and are rarely seen as their habitat is far underneath the ground. Their existence is however acknowledged by the presence of their mounds. In Malaysia, the *Thalassina* species have been found in remote localities such as the *T. anomala* in Penang and Sarawak (De Man 1928), Selangor (Sasekumar 1974) and Terengganu (Hassan *et al.* 2015) , the *T. kelanang* in Carey Island and Kuala Langat, Selangor (Moh & Chong 2009; Sasekumar 1974) and the *T. gracilis* in Kuala Langat, Selangor (Moh *et al.* 2013).

Mud lobsters are important organisms in the mangrove ecosystem due to its burrowing activities which help bring and recycle nutrients from underground to the upper levels of sediment (Kartika & Patria 2012). The digging activity by this species helps loosen the mud and allow air and oxygenated water to penetrate into the ground. Their nests provide habitat for many mangrove organisms such as the mud shrimp, mud crab, spiders, snakes, snails and flatworms (Macintosh *et al.* 2002).

In Malaysia and Thailand, mud lobsters are eaten by the local people as a traditional remedy for asthma. Over the years, it is traditionally believed that mud lobsters are able to improve asthma condition (Holthuis 1991; Hassan *et al.* 2015). According to Holthuis (1991), people in Thailand use the mud lobster as a remedy for asthma, although it is not eaten directly. Instead, mud lobsters are dried, grinded into powder form, and drunk with water or dissolved in alcoholic liquor for a couple of days before being drunk. However, the therapeutic potential of this crustacean has yet to be scientifically documented.

Asthma is a treatable but incurable chronic inflammatory disease characterised by a reversible airway obstruction (Murdoch & Lloyd 2010). Inflammatory mediators are released in allergic asthma and accompanied by inflammation of the airways with increased numbers of inflammatory cells accumulating in the alveolar submucosa (Vane & Botting 1987). Since mud lobsters have been used as a traditional medicine against asthma, present study was conducted to investigate the anti-inflammatory potential of three mud lobster extracts (hexane, chloroform and methanol) towards the lipopolysaccharide (LPS)-stimulated RAW 264.7 macrophages.

## MATERIALS AND METHODS

### Sample Collection, Species Identification and Sample Extraction

**Figure 1:**
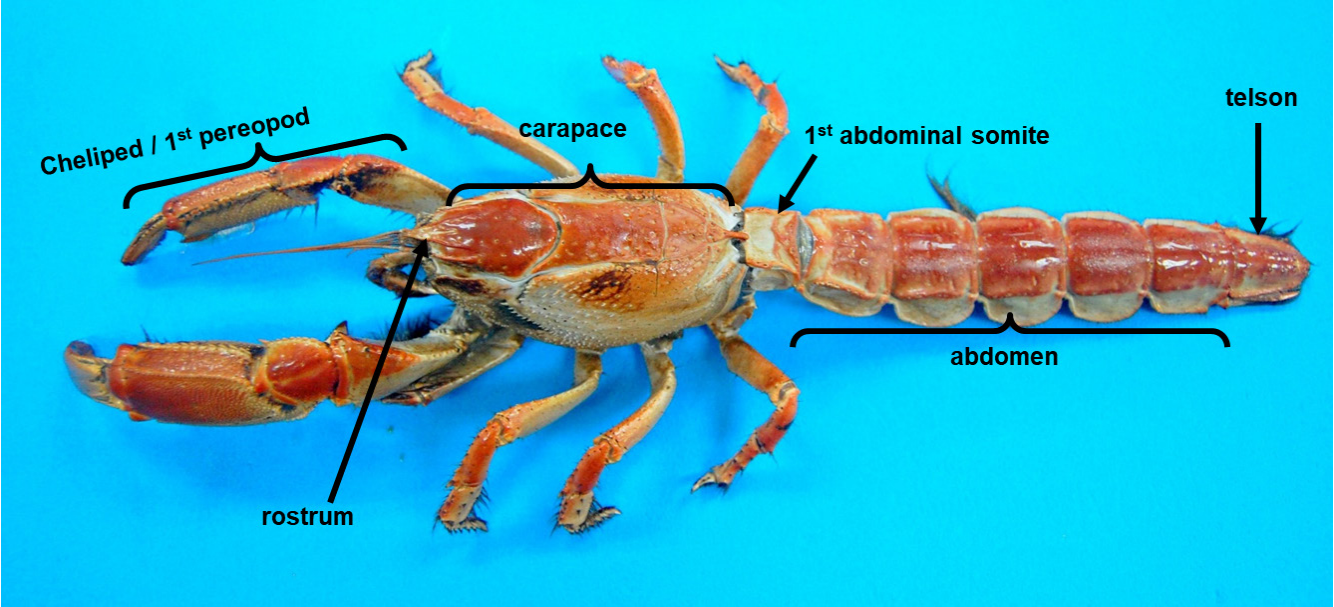
*Thalassina anomala* from Kuala Tatau, Bintulu, Sarawak, Malaysia.

Mud lobsters were purchased from local people in Kuala Tatau, Bintulu (3°05’42.8”N, 112°51’56.0”E), Kuala Balingian, Mukah (3°00’36.9”N, 112°35’26.0”E) and Sarikei (2°07’55.5”N, 111°30’59.8”E), Sarawak. The identification of species was carried out based on the morphological characteristics described in Moh and Chong (2009) and Moh *et al.* (2013). There are a few morphological features that can be examined to differentiate the species such as carapace, rostrum and abdominal somite (Fig. 1). Samples were transported to the laboratory and oven-dried at 50°C for 24 h until a constant weight was attained. The dried samples were then grinded into powder using an electric grinder. The mud lobster powder was macerated in hexane (MH), chloroform (MC) and methanol (MM) by using 2:5 ratio and vortexed for 30 s before placed in a 50°C water bath for 10 min. Next, the supernatants were centrifuged at 3000 rpm for 5 min and evaporated using a rotary evaporator at 40°C. The concentrated extracts of MH, MC and MM were freeze-dried and stored at 4°C until further investigation.

### Cell Culture

The murine macrophages cell line, RAW 264.7 was purchased from the American Type Culture Collection (ATCC, VA, USA) and cultured in Dulbecco’s Modified Eagle Medium (DMEM) supplemented with 10% fetal bovine serum (FBS), 1% streptomycin/penicillin at 37°C in a humidified incubator with 5% CO_2_. The cells were sub-cultured in 70%–90% confluent condition to ensure healthy propagation of the cell line.

### 3-(4,5-Dimethylthiazol-2-yl)-2,5-diphenyltetrazolium Bromide (MTT) Colorimetric Assay

RAW 264.7 macrophages were cultured in a 96-well plate (100 μL/well) at a density of 1 × 10^5^ cells/well and incubated for 24 h. The macrophages were treated with MH, MC and MM at various gradient concentrations of 15.625, 31.25, 62.5, 125, 250, 500 and 1000 μg/mL, followed by additional incubation for 24 h. Then, 20 μL of MTT solution (5 mg/mL) in phosphate-buffered solution (PBS) was added to each well. The macrophages were further incubated for 3 h. Next, the medium was removed and 100 μL of dimethyl sulfoxide (DMSO) was added to each well to dissolve the purple formazan crystals. The absorbance was measured using ELx800 Absorbance Microplate Reader (BioTek Instruments Inc., VT, USA) at a wavelength of 570 nm.

### Nitrite Quantification Assay

The effect of the mud lobster extract on the production of nitric oxide (NO) was determined using the level of nitrite accumulated in the culture medium. RAW 264.7 macrophages were cultured in 6-well plates at a density of 1 × 10^6^ cells/well with 2 mL of cell culture media. The macrophages were incubated for 24 h. The old medium was removed and replaced with fresh medium to maintain the cells. Next, the RAW 264.7 macrophages were treated with different concentrations of MH, MC, MM and a positive control, dexamethasone (0.5 μg/mL). This was followed with the induction of LPS (1 μg/mL) for all samples except in the control. The samples were further incubated for another 24 h. After incubation, 100 μL of collected cell culture medium was mixed with 100 μL of Griess reagent (0.1% NED, 1% sulphanilamide and 2.5% phosphoric acid) and incubated in dark condition at room temperature for 10 min. The absorbance was measured using ELx800 Absorbance Microplate Reader at a wavelength of 540 nm.

### Enzyme-linked Immunosorbent Assay (ELISA)

The RAW 264.7 macrophages were cultured in 6-well plates at a density of 1 × 10^6^ cells/well for 24 h. The macrophages were then treated with MH and dexamethasone (0.5 μg/mL), followed with the addition of LPS (1 μg/mL) to all macrophages except the control for another 24 h to induce inflammation. The concentration of PGE_2_ and cytokine mediators such as TNF-α, IL-6 and IL1β were assayed in the cultured media of macrophages using mouse ELISA kits (R&D Systems Inc., MN, USA) according to the manufacturer’s instruction.

### GC-MS Analysis

GC-MS analysis of MH was performed using GC-MS (Shimadzu QP2010 Ultra system, Kyoto, Japan) equipped with a capillary column Rxi-5ms (30 m length × 0.25 mm ID × 0.25 μm film thickness). The programmed temperatures were as follows: injector temperature was 250°C, ion-source temperature was 200°C, oven temperature was initially 50°C which was then increased at 3°C/min to 300°C and held for 10 min. Helium was used as the carrier gas at a pressure of 37.1 kPa. The mass spectra of separated components were identified based on data from WILEY and National Institute of Standards and Technology (NIST) libraries.

### Statistical Analysis

The results from three independent experiments were summarised and all data are expressed as the mean ± standard deviation (SD). The significant differences were examined using IBM SPSS 20.0 software (SPSS Inc., Chicago, USA), one-way analysis of variance (ANOVA) and Tukey’s post hoc test for pairwise comparison. *p*-values of 0.05 or less were considered as statistically significant.

## RESULTS

### Identification of Species

Based on the morphological characteristics described in Moh and Chong (2009), it was concluded that the samples collected are *T. anomala*. The features of the rostrum are useful when differentiating the species. The rostrum of all samples collected were triangular with a shallow median sulcus (groove) that does not extend beyond the adrostrals, properties identical to both *T. anomala* and *T. squamifera.* Meanwhile, *T. kelanang* has a waisted rostrum with an acute tip and a deep median sulcus (groove) that reaches behind the adrostrals. Besides, according to Moh and Chong (2009), the tergite of the first abdominal somite of *T. anomala* has two petaloid depressions in the form of an inverted V; these features existed in all of the samples collected. This differentiates *T. kelanang* and *T. squamifera* which have an inverted Y groove at the first abdominal somite located on the dorsal tergite.

### Effect of *T. anomala* Extracts on Cell Viability

**Figure 2:**
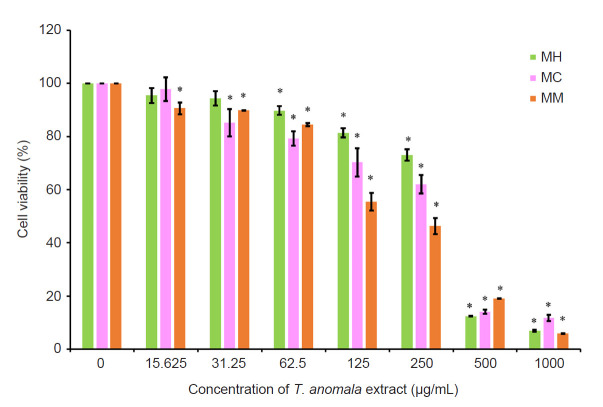
Effect of *T. anomala* extracts (hexane extract, MH; chloroform extract, MC and methanol extract, MM) on viability of RAW 264.7 macrophages.

The cytotoxicity effects of *T. anomala* extracts (hexan, MH; chloroform, MC and methanol, MM) on the RAW 264.7 macrophages were examined using MTT assay. As shown in Fig. 2, all extracts decreased the viability of the treated macrophages in a concentration-dependent manner. Concentrations of each extracts that exhibited more than 80% cell viability were chosen for further anti-inflammatory analysis. For MH, 25, 50 and 100 μg/mL concentrations were chosen. Meanwhile for MC, 12.5, 25 and 50 μg/mL concentrations were selected, and for MM, 15, 30 and 60 μg/mL concentrations were chosen for subsequent analysis.

### Effect of *T. anomala* Extracts on NO Production

**Figure 3:**
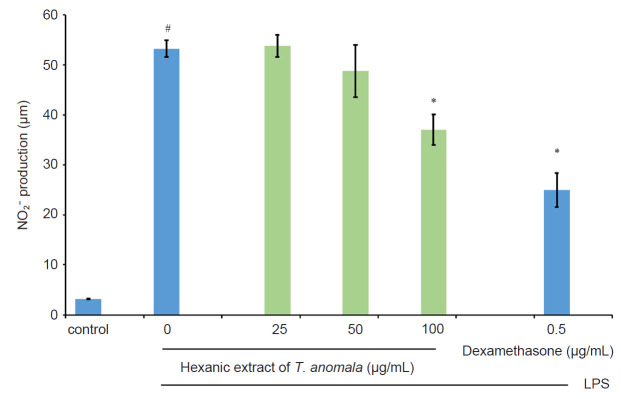
Effect of MH on NO production by LPS-induced RAW 264.7 macrophages.

**Figure 4:**
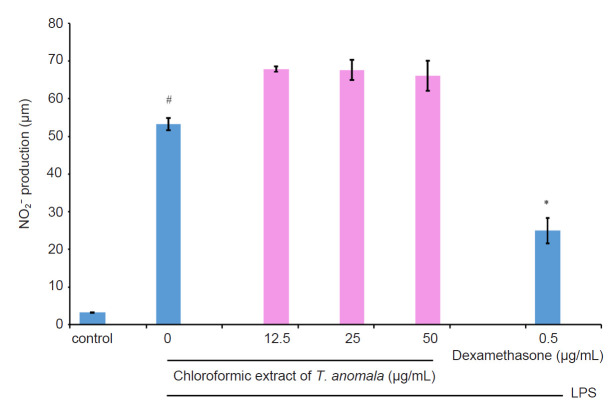
Effect of MC on NO production by LPS-induced RAW 264.7 macrophages.

**Figure 5:**
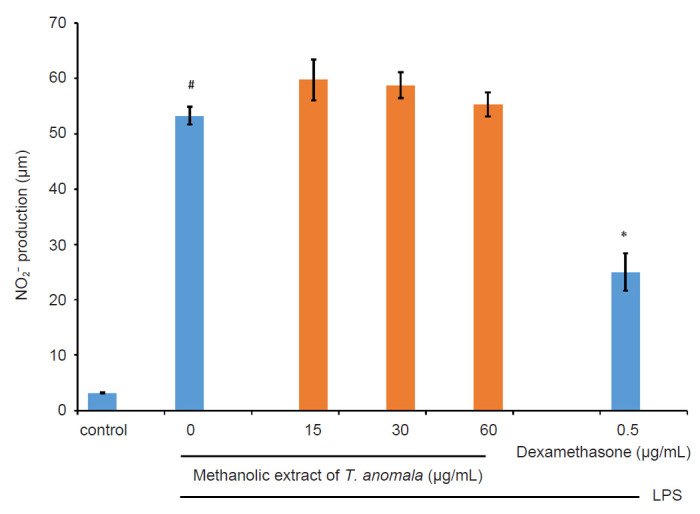
Effect of MM on NO production by LPS-induced RAW 264.7 macrophages.

The anti-inflammatory effect of *T. anomala* extracts (hexane, MH; chloroform, MC and methanol, MM) was evaluated by assessing the inhibition of NO production in LPS-induced RAW 264.7 macrophages by using the Griess reagent. The results from Figs. 3, 4 and 5 show that MH exhibits a good inhibitory effect on NO production compared to MC and MM. MC and MM did not show any inhibitory activity towards NO. Based on these results, MH was chosen for further anti-inflammatory analysis. The untreated control group released a low level of NO, while the LPS induction group without treatment elevated NO production (Fig. 3). In addition, dexamethasone as a positive control was able to suppress NO production significantly.

### Effect of Hexane Extract of *T. anomala* on PGE_2_ and Proinflammatory Cytokines Production (TNF-α, IL-6 and IL-1β)

**Figure 6:**
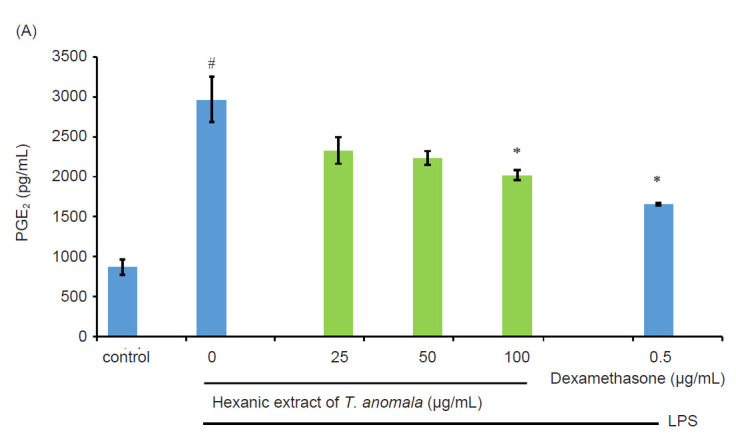

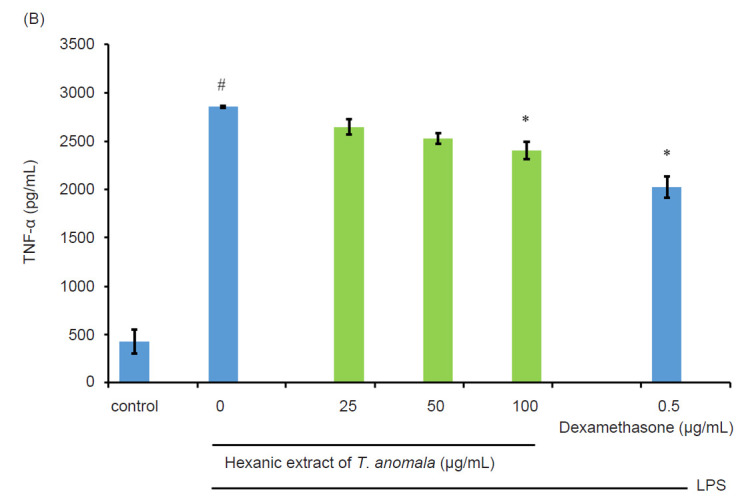

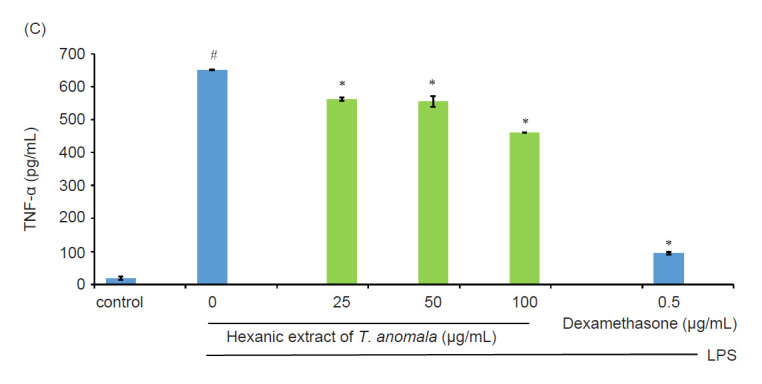

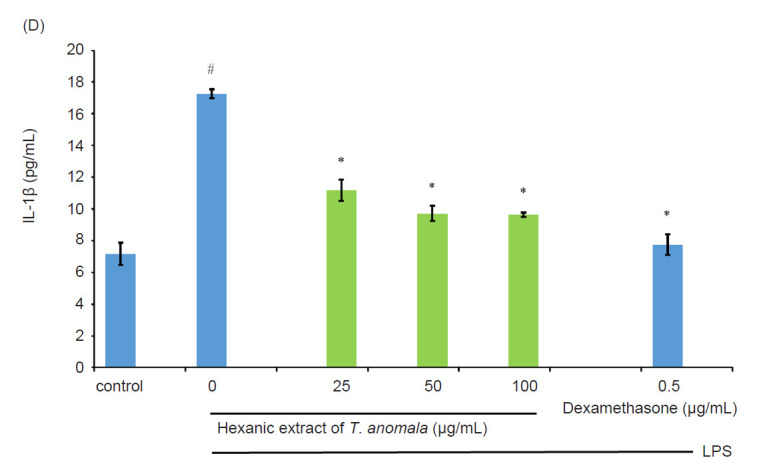
Effect of MH on the production of: (A) PGE_2_ by LPS-induced RAW 264.7 macrophages; (B) TNF-α by LPS-induced RAW 264.7 macrophages; (C) the IL-6 by LPS-induced RAW 264.7 macrophages; (D) IL-1β by LPS-induced RAW 264.7 macrophages.

Evaluation of anti-inflammatory activity was based on the ability of *T. anomala* extracts to suppress the production of PGE_2_ and proinflammatory cytokines (TNF-α, IL-6 and IL-1β) in LPS-induced RAW 264.7 macrophages. For this analysis, the hexane extract, MH was chosen as it was the only extract that exhibited anti-inflammatory activity in the NO analysis. Fig. 6A shows that the MH was able to significantly inhibit PGE_2_ production. Figs. 6B, 6C and 6D show that TNF-α, IL-6 and IL-1β productions were significantly elevated in response to LPS induction. However, upon treatment with MH, the levels of TNF-α, IL-6 and IL-1β were reduced significantly. Dexamethasone as a positive control was able to inhibit the production of PGE_2_ and proinflammatory cytokines as well.

### GC-MS Analysis of Active Compounds

The GC-MS analysis of the hexane extract of *T. anomala*, MH was conducted due to the presence of anti-inflammatory activity. The GC-MS analysis of MH revealed the presence of 19 putative compounds which account for its various biological activities including anti-inflammatory, antioxidant and anti-bacterial. The putative compounds along with their retention times, molecular formula, molecular weight and percentage of peak area are listed in Table 1.

## DISCUSSION

In this study, we observed three different extracts of *T. anomala* in order to investigate the organism’s anti-inflammatory potential through its ability to inhibit the production of NO, PGE_2_ and proinflammatory cytokines in LPS-stimulated RAW 264.7 macrophages. Among the three solvents, the hexane extract showed the highest inhibitory activity. A previous study done by Sindhu and Sherief (2011) reported anti-inflammatory activity among non-polar solvents extracts as well, such as the hexane:isopropanol fractions of Arabian red shrimp, *Aristeus alcocki*. In addition, a study done by Khanafari *et al.* (2007) showed that non-polar solvent extracts from the green tiger prawn, *Penaeus semisulcatus* exhibited astaxanthin, an anti-inflammatory compound (Farruggia *et al.* 2015).

Lipopolysaccharide (LPS) is a major component of the outer membrane of Gram-negative bacteria and acts as a potent initiator of inflammation (Meng & Lowell 1997). LPS activates monocytes and macrophages to produce pro-inflammatory mediators such as NO, PGE_2_ and cytokines (Raetz 1990; Guha & Mackman 2001; Cohen 2002). Hence, in the present study, LPS-stimulated RAW 264.7 macrophages were used as an in-vitro inflammation experimental model. The results showed increased levels of pro-inflammatory mediators observed in the LPS induction group without treatment.

The cytotoxicity effects of the three different extracts of *T. anomala* were determined using MTT assay. As shown in Fig. 2, the hexane extract showed the highest cell viability in comparison to the methanol and chloroform extracts. Concentrations of the extracts which are nontoxic to macrophages were chosen for further anti-inflammatory analysis. The non-toxic concentrations of the extracts were selected to ensure that the anti-inflammatory activity detected was caused by the genuine inhibitory effect of the compounds presented in the extract, and not due to cell death.

NO is a reactive free radical gas which plays a key role in regulating inflammatory responses. It is released in greater amounts during inflammation reactions (MacMicking *et al.* 1997). The overproduction of NO can lead to numerous inflammation-associated diseases such as cardiovascular disease, hypotension, vasodilatation, induction of apoptosis, and triggering of the pathogenesis of inflammation involving joints, guts and lungs (Sharma *et al.* 2007; Heo *et al.* 2014). Previous studies done by Alving *et al.* (1993); Kharitonov *et al.* (1995); Massaro *et al.* (1996) showed increased concentrations of exhaled NO in asthmatic subjects. Therefore, the level of NO may be a useful marker in monitoring asthma and evaluating the effect of anti-inflammatory treatments in reducing asthmatic symptoms (Kharitonov *et al.* 1994; Pendharkar & Mehta 2008). In the present study, treatments using hexane extract of *T. anomala* and a positive control drug, dexamethasone were able to supress the production of NO in LPS-stimulated RAW 264.7 macrophages in a concentration-dependent manner (Fig. 3).

Meanwhile, according to Tilley *et al.* (2003), PGE_2_ triggers the bronchoconstrictor effect via the EP1 and EP3 receptors. Previous studies had reported high levels of PGE_2_ in the sputum of patients with eosinophilic bronchitis, an airway inflammation disease that cause chronic cough (Brightling *et al.* 2000; Birring *et al.* 2004; Sastre *et al.* 2008). As shown in Fig. 6a, the PGE_2_ production in the LPS-stimulated macrophages was reduced after treatment with hexane extract of *T. anomala* by concentration dependently.

Several studies have reported that pro-inflammatory cytokines including TNF-α, IL-6 and IL-1β are involved in the pathogenesis of asthma as evidenced by their elevated levels in the sputum and bronchoalveolar lavage fluid of asthmatic patients (Kips *et al.* 1993; Albuquerque *et al.* 1998; Chung & Barnes 1999; Kips 2001; Mehta & Mahajan 2006; Barnes 2008). In the present investigation, a concentration-dependent decrease can be observed in the levels of TNF-α, IL-6 and IL-1β in LPS-stimulated RAW 264.7 cells after treatment with hexane extract of *T. anomala* (Figs. 6b–6d).

Based on the findings, the results suggest that the hexane extract of *T. anomala* has therapeutic potential as an anti-inflammatory agent to alleviate inflammatory respiratory ailments.

Identification of active compounds via GC-MS analysis revealed the presence of 19 putative compounds in the hexane extract of *T. anomala* as shown in Table 1. The major groups detected are cholesterol and fatty acids. According to Zagryagskaya *et al.* (2008), cholesterol exhibits anti-inflammatory activity and decreases the leukotriene synthesis in human polymorphonuclear leukocytes. Leukotriene is a substance released during asthma attacks and triggers bronchoconstriction (Berger 1999). Palmitoleic acid is another compound detected in the hexane extract of *T. anomala* that possess anti-inflammatory activity (Morse 2015; Astudillo *et al.* 2017). Studies done by Souza *et al.* (2017) reported that palmitoleic acid exhibits anti-inflammatory potential through its ability to reduce the expression of TNF-α and IL-6 in LPS-stimulated macrophages.

In addition, other compounds identified in the hexane extract of *T. anomala* that were reported to have anti-inflammatory activity are butyric acid (Załęski *et al.* 2013), isovaleric acid (Kim *et al.* 2014), hexadecanoic acid (Aparna *et al.* 2012), 2-Piperidinone (Li *et al.* 2016), indole (Hemalatha *et al.* 2013), squalene (Cárdeno *et al.* 2015), fumaric acid (Shakya *et al.* 2014), oleamide (Oh *et al.* 2010), 9-Octadecenoic acid methyl ester, hexadecanoic acid methyl ester and octadecanoic acid (Othman *et al.* 2015). Meanwhile, squalene and phenol, 2,5-bis(1,1-dimethylethyl) were reported to have antibacterial and antioxidant properties (Sermakkani & Thangapandian 2012; Manorenjitha *et al.* 2013).

## CONCLUSION

In conclusion, results obtained in this study suggested that the hexane extract of *T. anomala* exhibits anti-inflammatory potential in LPS-stimulated RAW 264.7 macrophages. The non-polar compounds detected in the hexane extract of *T. anomala* by GC-MS analysis revealed 19 putative metabolites which may contribute to the anti-inflammatory activity. The findings justify the traditional use of mud lobster as remedy for inflammation respiratory ailments.

## Figures and Tables

**Table 1:** The putative compounds detected in hexane extract of *T. anomala* by GC-MS analysis.

No.	R/T (min)	Name of the compound	Molecular formula	Molecular weight	Peak area %
1.	4.907	Butyric acid	C_4_ H_8_ O_2_	88	8.00
2.	6.068	Isovaleric acid	C_5_ H_10_ O_2_	102	2.88
3.	6.520	Isovaleric acid	C_5_ H_10_ O_2_	102	2.86
4.	19.253	2-Piperidinone	C_5_ H_9_ NO	99	3.55
5.	24.440	Indole	C_8_ H_7_ N	117	1.04
6.	34.134	Phenol, 2,5-bis(1,1-dimethylethyl)	C_14_ H_22_ O	206	1.29
7.	49.706	Hexadecanoic acid methyl ester	C_17_ H_34_ O_2_	270	0.84
8.	50.396	Palmitoleic acid	C_16_ H_30_ O_2_	254	3.42
9.	50.634	Cis-9-Hexadecenoic acid	C_16_ H_30_ O_2_	254	1.13
10.	51.090	Hexadecanoic acid	C_16_ H_32_ O_2_	256	4.78
11.	53.038	Octadecanoic acid	C_18_ H_36_ O_2_	284	0.42
12.	55.446	9-Octadecenoic acid methyl ester	C_19_ H_36_ O_2_	296	0.47
13.	57.280	Oleamide	C_18_ H_35_ NO	281	1.47
14.	60.403	Fumaric acid	C_17_ H_31_ NO_4_	313	0.64
15.	63.242	Oelic acid amide	C_18_ H_35_ NO	281	1.65
16.	63.419	Oleamide	C_18_ H_35_ NO	281	1.47
17.	65.954	Fumaric acid, 2-dimethylaminoethyl nonyl ester	C_17_ H_31_ NO_4_	313	0.98
18.	75.409	Squalene	C_30_ H_50_	410	0.32
19.	82.451	Cholesterol	C_27_ H_46_ O	386	25.07
